# A Soft-Fault Diagnosis Method for Coastal Lightning Location Networks Based on Observer Pattern

**DOI:** 10.3390/s25154593

**Published:** 2025-07-24

**Authors:** Yiming Zhang, Ping Guo

**Affiliations:** School of Computer Science, Nanjing University of Information Science and Technology, Nanjing 210044, China; 202283330026@nuist.edu.cn

**Keywords:** coastal lightning location network, observer pattern, soft-fault diagnosis, wireless sensor network

## Abstract

Coastal areas are prone to thunderstorms. Lightning strikes can damage power facilities and communication systems, thereby leading to serious consequences. The lightning location network achieves lightning location through data fusion from multiple lightning locator nodes and can detect the location and intensity of lightning in real time. It is an important facility for thunderstorm warning and protection in coastal areas. However, when a sensor node in a lightning location network experiences a soft fault, it causes distortion in the lightning location. To achieve fault diagnosis of lightning locator nodes in a multi-node data fusion mode, this study proposes a new lightning location mode: the observer pattern. This paper first analyzes the main factors contributing to the error of the lightning location algorithm under this mode, proposes an observer pattern estimation algorithm (OPE) for lightning location, and defines the proportion of improvement in lightning positioning accuracy (PI) caused by the OPE algorithm. By analyzing the changes in PI in the process of lightning location, this study further proposes a diagnostic algorithm (OPSFD) for soft-fault nodes in a lightning location network. The simulation experiments in the paper demonstrate that the OPE algorithm can effectively improve the positioning accuracy of existing lightning location networks. Therefore, the OPE algorithm is also a low-cost and efficient method for improving the accuracy of existing lightning location networks, and it is suitable for the actual deployment and upgrading of current lightning locators. Meanwhile, the experimental results show that when a soft fault causes the observation error of the node to exceed the normal range, the OPSFD algorithm proposed in this study can effectively diagnose the faulty node.

## 1. Introduction

Owing to its open topography and landforms, the coast is vulnerable to the influence of strong convective weather, especially thunderstorm activities. Thunderstorms can cause overvoltage in coastal power systems, leading to serious consequences [1]. The strong electromagnetic field generated by lightning may also interfere with communication signals and damage marine communication systems [2]. The lightning location network, by observing the process of lightning occurrence and the spatial position of lightning in real time, predicts the evolution trajectory of lightning weather, thereby enabling the early shutdown of equipment or the activation of protective devices. This is of great significance for safeguarding coastal power and electronic systems. At present, most countries have deployed lightning location networks in their coastal areas to monitor lightning activity and prevent the risk of lightning strikes [3,4].

**Figure 1:**
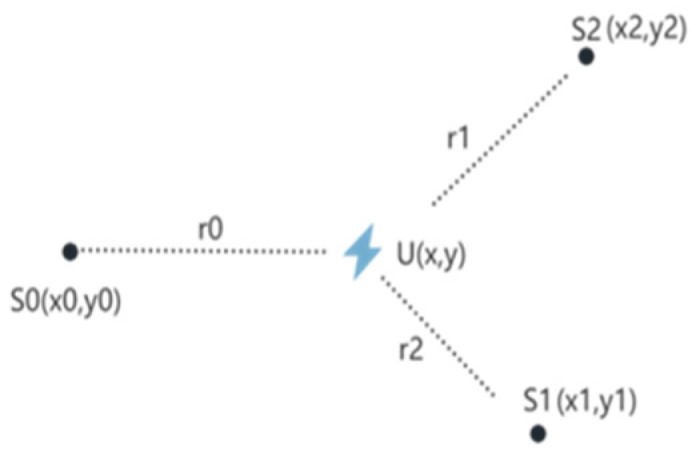
Organization model diagram of lightning location network.

A lightning location network is a wireless sensor network composed of multiple lightning signal sensors (lightning location devices), and its organizational model is shown in Figure 1.

As shown in Figure 1, to achieve spatial positioning of lightning, a lightning location network is composed of at least three (generally more than three) lightning locators. When lightning occurs within the observation range of a lightning locator, the instrument records the arrival time of the lightning signal and sends it to the data center of the lightning location network. When the data center of the lightning location network receives the observation data of the same lightning from multiple lightning locator nodes, it fuses observation data and calculates the time difference of the arrival of the lightning signal observed by each node. Then it uses a positioning algorithm to estimate the longitude and latitude of the lightning occurrence location.

The lightning location algorithm is the core for realizing the functions of the lightning location network. Currently, the main algorithms for lightning location include Time Difference of Arrival (TDOA) [5], Multi-Directional Frequency (MDF) [6], and Frequency Difference of Arrival (FDOA) [7]. Among them, TDOA achieves the positioning of lightning based on the time difference of the lightning information observed by each positioning instrument, whereas MDF and FDOA are technologies that use the electrical (magnetic) direction finding of lightning for positioning. Each of these methods has advantages and disadvantages. Owing to the numerous advantages of lightning locators using TDOA technology in terms of cost, deployment convenience, and anti-interference capabilities [8], most lightning location networks are based on TDOA or combine TDOA with other algorithms to determine lightning location [9].

Regardless of the positioning algorithm used, a lightning location network needs to fuse the observation data from multiple lightning locators to determine the lightning location. If a node in the lightning location network malfunctions and the observed data deviate significantly from the true value, such errors spread through the location calculation equation and affect the final lightning location result, causing the lightning location of the entire network to be distorted.

In general, a data center maintains periodic communication with lightning locator nodes. When a hard fault occurs in the lightning locator node (such as a power failure, module malfunction, or communication failure), the data center can promptly detect and handle it through the communication status with the node. However, when a soft fault occurs in the lightning locator node (abnormal observation data causing observation errors to exceed the allowable range), since the node can still normally send and receive data, the center cannot promptly identify the node with the soft fault. Consequently, for a long period of time, the data availability of the lightning location network is affected, which seriously affects the safety and reliability of coastal power and electronic systems.

At present, the main approaches to implementing soft-fault diagnosis technology for wireless sensor network nodes include diagnosing the node’s own status, diagnosing based on the comparison of the node’s status with that of adjacent nodes, and diagnosing by comparing the historical data of the observed target with the current data. These diagnostic techniques include methods based on statistical models, methods based on machine learning, and methods based on deep neural network learning.

The above-mentioned method cannot fully adapt to the observation and location of coastal lightning because each location instrument node in the lightning location network works independently and does not exchange data. Moreover, most existing location instrument nodes do not have the function of monitoring their own working status. Additionally, lightning is a one-off instantaneous weather process, and it is impossible to diagnose nodes by comparing historical observational data. Furthermore, because the results of lightning location are derived from the fusion of sensor data from multiple lightning location devices, the aforementioned diagnostic methods cannot be fully applied to lightning location scenarios.

In response to the above issues, this paper proposes a soft-fault diagnosis method for coastal lightning location networks based on the observer pattern. The main contributions of this study are as follows:

1. A lightning location network model is proposed based on the observer pattern: This study proposes a lightning location network model using the observer pattern, where the observer refers to a high-precision lightning locator in a lightning location network. The observer pattern means that in the lightning location network, the high-precision lightning locator is used as the main station, combined with ordinary lightning locators to form a lightning location network. Owing to the long-term construction and development of lightning locator networks, a deployment pattern has been formed where lightning locators with different specifications and models are mixed. Under the observer pattern, a lightning location network can integrate data from various types of lightning locators. The simulation experiments in this study demonstrate that the observer pattern can effectively improve the accuracy of the lightning location.
2. Based on the observer pattern, the OPE lightning location algorithm is proposed: This study first analyzes the main factors contributing to the error of the lightning location algorithm under the observer pattern, and then proposes an estimation algorithm for lightning location based on the observer pattern (OPE). Compared with other similar TDOA algorithms [5], such as Chan [10,11], WLS (weighted least squares) [12], and ML (maximum likelihood) [13], and positioning algorithms implemented using deep learning [14], the simulation experiments in this study show that using a single high-precision node as an observer node can effectively improve the accuracy of the entire lightning location network. Therefore, the OPE algorithm proposed in this paper also provides an effective and low-cost way to upgrade existing lightning location networks, making it more suitable for current deployment and application scenarios of lightning locators.
3. Diagnostic algorithm for soft faults of lightning location nodes based on the OPE algorithm: This paper analyzes the proportion of improvement in lightning positioning accuracy (PI) achieved under the observer pattern. Furthermore, we analyze the variation in PI when soft-fault nodes exist in the lightning location network. Then this study presents a diagnostic algorithm for soft-fault nodes in a lightning location network. Simulation experiments show that the method proposed in this study can diagnose common positioning instrument nodes with soft faults, which is of significant importance for ensuring the data availability of the lightning positioning instrument network.

The remainder of this paper is organized into four parts. Firstly, it will introduce the research background and related work of this article, mainly including the fault diagnosis methods of wireless sensor networks, etc. The second part introduces the observer pattern proposed in this study and presents the main components of lightning location errors under the observer pattern. The third section introduces the algorithm and principle of this study. By fusing the measurement data of lightning information from observer nodes and ordinary positioning instrument nodes, an estimation algorithm for the location of lightning occurrence under the observer pattern is presented: the OPE algorithm. Based on the OPE algorithm, the lightning locator soft-fault node diagnostic algorithm proposed in this study was further developed. The last part presents the simulation experiment results, analysis, and summary of this study.

## 2. Related Work

### 2.1. Soft-Fault Model of Lightning Locator Node

Owing to the large area of the coastal region, lightning location networks deployed in coastal areas usually transmit data through wireless communication. Therefore, these lightning location networks are wireless sensor networks. Because the lightning location network is composed of lightning locator nodes, wireless networks, and data centers, the location where faults occur may be either at the lightning locator nodes, in the wireless data transmission network, or in data centers.

As the data center of the lightning location network maintains periodic communication with the lightning locator nodes at all times, any transmission faults in the wireless data network are detected immediately by the data center. Because the data center is usually staffed, any faults in the data center will also be promptly discovered. Some lightning locator nodes are deployed in the wild without human supervision. Therefore, the fault diagnosis of lightning location networks mainly focuses on the fault diagnosis of lightning locator nodes.

As mentioned in reference [15], faults occurring in lightning location network nodes can be classified into hard and soft faults, among which are the following:

Hard faults refer to non-self-repairable faults caused by hardware modules or the power supplies of nodes. Such faults lead to the loss of the node’s working ability, thus preventing it from sending sensor data normally.

Soft faults refer to the distortion of sensor data caused by various reasons in nodes (excluding occasional recoverable transient faults but including long-term distortion of sensor data), and such faults cause nodes to continuously send abnormal sensor data. Therefore, when sensor nodes experience soft faults, they will cause distortion of sensing data, thereby leading to data availability issues for the entire system and even triggering serious consequences [15]. The objective of this study is to diagnose the soft faults of sensor nodes in a lightning location network.

According to the soft-fault modeling of sensor nodes in reference [16], there are a total of six types: bias faults, which refer to a fixed deviation value between the sensor data and the true data; gain faults, which refer to the ratio of the sensor data to certain true data; fixed value faults, where the sensor data are always certain values; out-of-bound faults, where the sensor data exceed the allowed range of values; peak faults, where the variation range of the sensing data exceeds the allowable range; and data loss faults, where data is lost during the data transmission of a node with this type of fault.

Because the object of observation for the lightning location network is lightning, and the location where lightning occurs is random, it is easy to diagnose the lightning locator nodes with fixed-value faults. Moreover, lightning is a transient meteorological process, and there are no continuous observations; so, there are no out-of-bound faults or peak faults for lightning locator nodes. As mentioned earlier, lightning locator nodes with data loss faults can also be easily diagnosed during the communication process with a data center.

Therefore, the soft faults of lightning locator nodes mainly include offset and gain faults. As a sensor, the lightning locator node has a certain observation error. Additionally, the observation error of a lightning location system is expressed by a normal distribution, that is $E\sim N(\mu ,\sigma^{2})$. Under normal conditions, the lightning locator follows the expected normal distribution parameters. However, when a lightning locator experiences a soft fault, the distribution of its observation errors deviates from the expected distribution. Therefore, this study assumes the following two situations as the soft faults of the lightning locator nodes to be diagnosed:

The error of the soft-fault node observing the flash signal follows the distribution of $E\sim N(\mu ,\sigma^{2})$. where $\sigma >\sigma_{0}$, and $\sigma_{0}$ is the variance of the observation error for normal nodes;

The error of the soft-fault node observing the flash signal follows the distribution of $E\sim N(\lambda ,\sigma^{2})$, where λ>μ; soft faults of the lightning locator node set in this study simultaneously include the sensor node offset and gain faults mentioned above.

### 2.2. Data-Oriented Soft-Fault Diagnosis Technology

According to the location where the diagnostic methods are executed, existing soft-fault diagnosis methods for wireless sensor nodes can be generally classified into three categories: centralized, distributed, and hybrid [15]. Centralized refers to the execution of sensor node diagnosis at the network center or data center, while distributed is carried out by the sensor nodes themselves, and the hybrid is executed in collaboration between the network center and nodes. Because the nodes of the general lightning location system are meteorological observation instruments and do not have the function of self-status detection, the diagnosis of soft faults of the nodes can only be carried out by the data center of the location network. Therefore, this study adopted a centralized diagnosis method.

Research on centralized diagnosis can be generally divided into two categories: one is to achieve diagnosis based on the data transmission status of nodes, and the other is to conduct analysis and diagnosis based on the sensor data of nodes. As the data center of the lightning location network can monitor the communication status of the location instrument nodes at any time, it can detect nodes with data transmission faults. Therefore, this study adopted the diagnosis of soft-fault nodes based on node-sensing data.

At present, research on diagnosing soft-fault nodes through node sensing data essentially regards the diagnostic process as a classification problem, that is, to distinguish normal nodes from soft-fault nodes through classification.

Early research in this area was conducted using statistical methods to achieve soft-fault diagnosis of nodes. Diagnostic methods for such studies are based on Z-value statistics. For instance, as assumed in [17], if the measurement error follows a normal distribution, the mean and variance of the sensor data can be statistically analyzed, and the Z-test can be used to diagnose faulty nodes. Specifically, if the absolute value of the measurement error of a node exceeds three times the variance, the node is regarded as faulty. There are also related works, such as [18], around Z-value statistics. They calculated the median of each partition by partitioning the nodes, and determined whether a node was faulty based on whether its sensor data exceeded the maximum median. Similar studies include [19] and others.

In contrast, [20] employed the Mann–Whitney U statistical test for soft faults. Meanwhile, reference [21] utilized the Kullback–Leibler divergence to analyze the degradation of sensor accuracy and diagnose soft faults. Reference [22] proposed the MIV algorithm, which analyzes the correlation of collected data between sensor nodes and their neighboring nodes. The fault diagnosis of the nodes is achieved by calculating the mutual information (MI) between the sensor data and comparing the MI under normal conditions with that under the current state.

With the rise of soft computing technologies, such as machine learning and deep learning, research on diagnosis based on soft computing has also been increasing. Among the methods frequently applied in such work are the Support Vector Machine (SVM), Convolutional Neural Network (CNN), Random Forest (RF), multilayer perceptron (MLP), Stochastic Gradient Descent (SGD), and deep learning, as shown in [23,24].

The above-mentioned statistical diagnostic methods mainly achieve the diagnosis of soft faults in nodes by statistically analyzing the distribution of relevant parameters of the target node or nearby nodes under normal conditions and using this as a reference. However, in the context of coastal lightning location networks, most lightning location devices are deployed at considerable distances from each other, and lightning is an instantaneous meteorological phenomenon that cannot be tracked continuously. Therefore, it is difficult to adapt the aforementioned methods directly to the research objectives of this study.

Methods based on soft computing technology require a large number of training samples, which are difficult to obtain. However, some of these methods lack theoretical derivations and explanations for diagnostic results.

In response to the above issues, this paper proposes an observer pattern based on the combination of different types of lightning locators and realizes the location of soft-fault nodes through the analysis of lightning location data under the observer pattern.

## 3. Diagnosis of Soft-Fault Lightning Locator Under Observer Pattern

### 3.1. Introduction to Lightning Positioning Algorithms

In the following, this paper first introduces the lightning positioning algorithms. When lightning occurs within the range of a lightning locator S_i_, the time when S_i_ observes the lightning is Equation (1):

(1) $$t_{i}=T+\sqrt{(x-x_{i}{)}^{2}+(y-y_{i}{)}^{2}}/c.$$

It can be seen from Formula (1) that the observation time of the lightning signal by the lightning locator is mainly composed of two parts, where $\sqrt{(x-x_{i}{)}^{2}+(y-y_{i}{)}^{2}}/c$ is the time required for the lightning signal to reach the location of lightning locator (*x_i_*, *y_i_*) from the location where lightning occurs (*x*, *y*), and *c* is the speed of light; *T* is the time when the lightning happens. Therefore, t_i_ represents the moment when the lightning signal reaches the lightning locator.

According to positioning in two-dimensional space, at least three lightning positioning instruments are required to achieve the positioning of lightning. After the data center receives the lightning observation time values *t_i_* sent by each lightning location node, it selects one node as the main station (assuming that the main station is U in Figure 1), and the other nodes as auxiliary stations. The distance difference of the lightning signal observed by the main station and auxiliary stations is calculated as Equation (2). Since *T* in Equation (1) is the time of lightning occurrence, it is the same for any locator node that observes the lightning signal, so the T part of Equation (1) is not included in Equation (2):

(2) $$r_{i0}=\sqrt{(x-x_{i}{)}^{2}+(y-y_{i}{)}^{2}}-\sqrt{(x-x_{0}{)}^{2}+(y-y_{0}{)}^{2}}.$$

According to the observation distance difference between the master station and multiple auxiliary stations, equations can be constructed to calculate the location of lightning. At present, the TDOA lightning location algorithms include the Chan algorithm, WLS algorithm, and ML algorithm. Among them, the Chan algorithm is the most widely used. The principle of the Chan algorithm is as follows.

According to Equation (2) and *k_i_* = *x_i_*^2^ + *y_i_*^2^, the following Equation (3) can be obtained:

(3) $$\begin{array}{ll} r_{i}^{2}-r_{0}^{2} & =(x-x_{i}{)}^{2}+(y-y_{i}{)}^{2}-(x-x_{0}{)}^{2}+(y-y_{0}{)}^{2}\\ & =x_{i}^{2}+y_{i}^{2}-2xx_{i}-2yy_{i}-(x_{0}^{2}+y_{0}^{2}-2xx_{0}-2yy_{0})\\ & =k_{i}-k_{0}+2x(x_{0}-x_{i})+2y(y_{0}-y_{i}) \end{array}$$

Since *r_i_*, *r*_0_, *k_i_*, and *k*_0_ are known, the equations can be constructed by two auxiliary stations and the main station, and the following Equation (4) can be obtained, where *x_i_*_0_ = *x_i_* − *x*_0_, and $d_{i}=\frac{k_{i}-k_{0}+r_{0}^{2}-r_{i}^{2}}{2}$:

(4) $$\left[\begin{array}{cc} x_{10} & y_{10}\\ x_{20} & y_{20} \end{array}\right]\left[\begin{array}{c} x\\ y \end{array}\right]=\begin{array}{c} d_{1}\\ d_{2} \end{array}$$

By solving Equation (4), the location of lightning occurrence can be realized. In addition, the WLS algorithm introduced a weight matrix on the basis of the Chan algorithm, and estimated the target position by the weighted least-squares method. However, the ML algorithm estimates the target position by maximizing the likelihood function, assuming that the measurement noise follows a known distribution (such as a Gaussian or exponential distribution), and is able to achieve the Cramer-Rao Lower Bound (CRLB). Due to space limitations, this paper will not discuss the derivation process of these two algorithms.

### 3.2. Observer Pattern and Analysis of Lightning Location Error Under Observer Pattern

**Figure 2:**
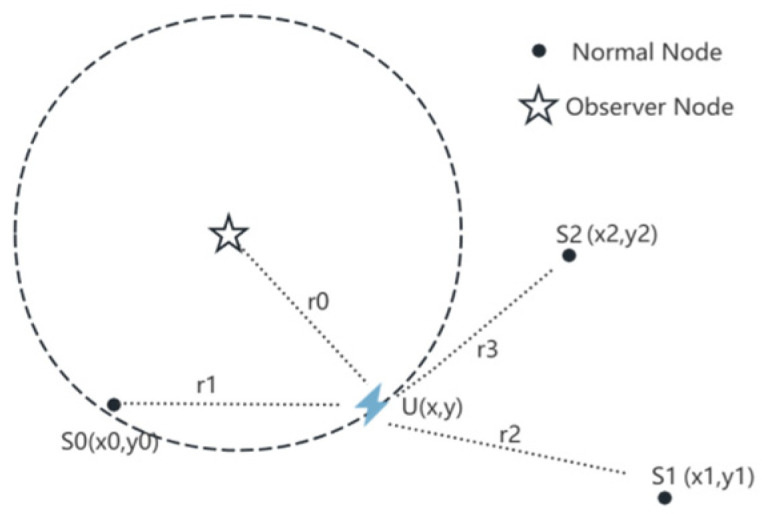
Geometric model diagram of the observer pattern.

With the development of microelectronics and signal processing technologies, the new generation of high-precision lightning locators has a much higher observation accuracy for lightning signals than ordinary lightning locator nodes [25,26]. Because the price and deployment cost of high-precision lightning locators are higher than those of ordinary lightning locators, current coastal lightning location networks tend to adopt a mixed deployment form of a small number of high-precision lightning locators and a large number of ordinary lightning locators. For such a deployment form, the observer pattern proposed in this study is shown in Figure 2, where the observer refers to a new type of high-precision lightning locator node.

Under the observer pattern, the high-precision lightning locator acts as an observer and serves as the main station (indicated by the star in the Figure 2), participating in the lightning location network composed of ordinary nodes to determine lightning location. In Figure 2, U represents the spatial location where lightning occurs, with its two-dimensional coordinates being (*x*, *y*). S0, S1, and S2 are the three lightning location sensor nodes and their two-dimensional coordinates are (*x*_0_, *y*_0_), (*x*_1_, *y*_1_), and (*x*_2_, *y*_2_), respectively.

As shown in the observer pattern structure in Figure 2, a lightning location network is generally composed of multiple nodes. The accuracy of the lightning location results is affected by the observation errors of each node. To minimize the impact of node observation errors on the location results as much as possible, a series of TDOA algorithms has been developed through long-term improvement and optimization. Essentially, these algorithms were implemented based on the least-squares method. In the following, we analyze the error composition under the observer pattern.

To evaluate the impact of the observation error at the lightning location node on the positioning result of the lightning signal, the derivative of Equation (2) was calculated, leading to Equation (5):

(5) $$dr_{i0}=(\frac{x-x_{i}}{r_{i}}-\frac{x-x_{0}}{r_{0}})dx+(\frac{y-y_{i}}{r_{i}}-\frac{y-y_{0}}{r_{0}})dy+(k_{0}-k_{i})$$

where $k_{i}=\frac{x-x_{i}}{r_{i}}dx_{i}+\frac{y-y_{i}}{r_{i}}dy_{i}$.

From Equation (5), by combining the derivative equations of the distance differences between multiple secondary stations and the main station, the matrix equation can be obtained as follows:

(6) dR=H•dX+dS.

(7) $$H=\left[\begin{array}{c} \begin{array}{cc} \frac{x-x_{1}}{r_{1}}-\frac{x-x_{0}}{r_{0}} & \frac{y-y_{1}}{r_{1}}-\frac{y-y_{0}}{r_{0}}\\ \frac{x-x_{2}}{r_{2}}-\frac{x-x_{0}}{r_{0}} & \frac{y-y_{2}}{r_{2}}-\frac{y-y_{0}}{r_{0}} \end{array}\\ \begin{array}{cc} ... & ...\\ \frac{x-x_{n}}{r_{n}}-\frac{x-x_{0}}{r_{0}} & \frac{y-y_{n}}{r_{n}}-\frac{y-y_{0}}{r_{0}} \end{array} \end{array}\right].$$

It is noted that in the matrix equation of Equation (6), the dX part involves the differential of the lightning location result, whereas the dS part only involves the differential of the node position of the lightning locator. Therefore, the influence of node observation errors on lightning location can be divided into two parts: dX is the lightning location error and dS is the error caused by the position of the location node.

It is assumed that the signal observation errors of each lightning locator node and deployment position errors follow independent normal distributions. Through further analysis, the influence of the observation errors of each node on the final lightning location can be determined, thereby calculating the GDOP (Geometric Dilution of Precision) [27] distribution of each node.

Further, assume that in the observer pattern, the observation error of a normal ordinary node obeys $E\sim N(\mu ,\sigma^{2})$, whereas the observation error of an observer node obeys $E\sim N(\mu_{0},\sigma_{0}^{2})$. Since the accuracy of the observer node is much higher than that of the ordinary node, *μ* ≫ *μ*_0_ and σ ≫ $\sigma_{0}$; also, because the working states of the observer node and the ordinary node are independent, the time difference *r_i_*_0_ of the lightning signal reaching the ordinary node and the observer node is defined in Equation (8),where $r_{i}^{*}$ and $r_{0}^{*}$, respectively, represent the true values of the lightning signal observation:

(8) $${r_{i}}_{0}=r_{i}^{*}-r_{0}^{*}+N(\mu -\mu_{0},\sigma^{2}+\sigma_{0}^{2}).$$

In addition, because μ ≫ μ_0_ and σ ≫ $\sigma_{0}$, $\mu -\mu_{0}\approx \mu$, $\sigma^{2}+{\sigma_{0}}^{2}\approx \sigma^{2}$; then, $d(r_{i0})\sim N(\mu ,\sigma^{2})$.

Equation (8) indicates that after introducing the observer node as the master station, the lightning signal observation errors between each ordinary lightning locator node and observer node are approximately equal to their own observation errors.

Meanwhile, by analyzing Equation (6) in the above, considering the application scenarios of lightning positioning, the deployment positions of lightning positioning instruments use GPS positioning or China’s Beidou positioning. The positioning accuracy of the deployment positions of the positioning instruments is generally at the meter level. However, the accuracy of observing lightning signals using ordinary lightning positioning instruments is generally at the kilometer level. Even for high-precision lightning positioning instruments, their observation accuracy is in the order of tens of meters. Therefore, in the application scenarios of lightning positioning, the station site error has a relatively small impact on the lightning positioning accuracy, and error Formula (6) can be further simplified as Equation (9):

(9) dR=H•dX.

Equation (9) shows that after obtaining the positioning error of a certain lightning strike, the positioning error of the i-th lightning strike can be calculated as Equation (10):

(10) $$dR_{i}=[dr_{10},dr_{20},...,dr_{no}]$$

Therefore, if the accurate location of the lightning occurrence can be obtained and the lightning location error can be calculated, the observation errors of each common locator node can be derived. Thus, the above analysis provides a theoretical basis for the soft-fault diagnosis of lightning location nodes based on the observer pattern.

### 3.3. Estimation Algorithm of Lightning Location Under the Observer Pattern

As analyzed above, to obtain the observation errors of each ordinary positioner, it is necessary to first obtain accurate lightning positioning. In particular, when there are already soft-fault nodes in the lightning location network, the lightning positioning obtained by the Chan series algorithm cannot be used to accurately position the lightning. Therefore, this paper proposes the use of an observer as the main station for lightning positioning to realize an estimation algorithm: Algorithm 1 (OPE) for lightning positioning.

Generally, due to the high construction cost of observer nodes and the difficulty in large-scale deployment, in order to improve the adaptability of the diagnostic algorithm in this paper, this paper assumes that in the observer pattern, only one observer node is involved in lightning location, as shown in Figure 2.
**Algorithm 1:** OPE: Estimation algorithm of lightning location based on observer pattern**Input:**
    Observer node position: (X_0_, Y_0_)
    Observed lightning distance from the observer: R_0_
    Set of observed distances from n ordinary nodes: S = {R_1_, R_2_, …, R_n_}
**Output:**

    Estimated lightning location (X, Y)
**Steps:**

**Initial Estimation via Chan Algorithm:**Compute the preliminary lightning location L′ = (X′, Y′), using the Chan algorithm on the distance set S;**Initialize Candidate Point Set:**Generate candidate points (X*, Y*) lying on the circle centered at (X_0_,Y_0_) with radius R0 (Discretize a circle into N uniformly spaced points for computational feasibility.)**Compute Average Observation Error:**For each candidate point L = (X*, Y*) in candidate point set
    3.1**Assume Exact Location:** Assume that L is the exact lightning location.    3.2**Calculate Positional Deviation**: Obtain the deviation e between L and L′: e = (X* − X′, Y* − Y′)    3.3**Calculate the observation error for ordinary nodes:** According to equation 10, get each ordinary node’s observation error: dR_10_, dR_20_, …, dR_n0_    3.4**Calculate Error Variance:** $\sigma^{2}=\sum_{i=1}^{n}{\left(dR_{i0}-\bar{dR}\right)}^{2}$**Select Optimal Estimate:**Identify the candidate point (X, Y) with the minimal error variance, output as the final estimated lightning location.


Suppose that during a certain lightning event, the observer node observes the occurrence distance of the lightning as r_0_. Then, the precise location of this lightning occurrence should be on a circle with the observer node as the center and a radius of r_0_. Let the precise location of the lightning be a point (x,y), and the location of this lightning by the common positioning instrument node network be $\left(x^{*},y^{*}\right)$; then, the position difference between the two location points is dX, and $dX=[x^{*}-x,y^{*}-y]$.

According to Equation (10), for this lightning, it can be obtained that the observation errors of each ordinary positioning instrument node are $dr_{10},dr_{20},...,dr_{no}$. Moreover, since the lightning positioning errors generated by each lightning positioning instrument node are independently and identically distributed, when all nodes are under normal conditions, the errors of each node $dr_{10},dr_{20},...,dr_{no}$ should also follow the $E\sim N(\mu ,\sigma^{2})$ distribution. Assuming that all ordinary lightning positioning instrument nodes are in a normal state and that each ordinary node is independently and identically distributed, the mean of these observation errors should be approximately equal to μ, and the variance is subject to $\sigma^{2}$.

From this, the estimation algorithm (OPE) proposed in this paper can be obtained as follows:

From the above OPE algorithm, it can be seen that the OPE algorithm is based on the Chan series algorithm for estimating the precise location of lightning occurrence.

**Figure 3:**
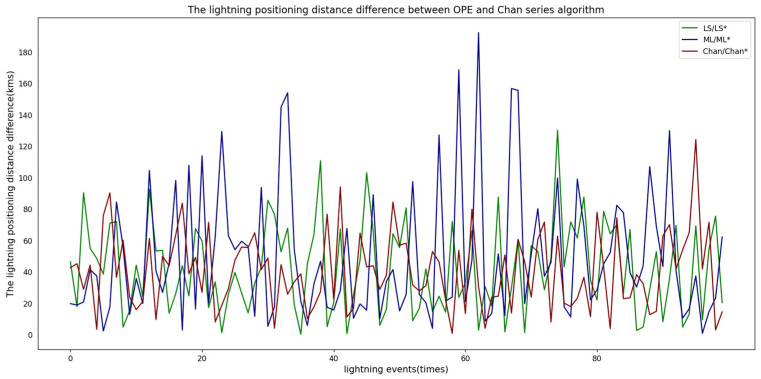
The positioning distance differences of three different Chan algorithms and their OPE algorithms. (In the figure, LS/LS*, ML/ML*, Chan/Chan* respectively represent the differences between the estimated positioning based on different algorithms applying the OPE algorithm and the original algorithm positioning).

In this study, first, on the circle generated with the observer node as the center and the distance R of the observed lightning signal as the radius (as shown in Figure 3), N points were evenly divided. We select one point L and assume that this point is the precise location of lightning. The observation error of each common node can then be calculated according to Equation (10), and the variance of the observation errors of each node is calculated.

Under normal circumstances, the observation errors at each node in the lightning locator network follow a unified and independent normal distribution. Therefore, the variance in dRi should be minimized. Therefore, in this study, the OPE algorithm selected the candidate point that minimized the variance value of dRi as the final lightning location estimation value.

### 3.4. Diagnostic Algorithm of Soft-Fault Nodes in the Observer Pattern

As mentioned earlier, a soft fault refers to a situation in which various reasons cause a significant deviation in the observation results of lightning signals by the lightning positioning instrument node. Under normal circumstances, the error in the lightning positioning node for the lightning observation result follows a normal distribution, that is $E\sim N(\mu ,\sigma^{2})$.

As described above, the following two situations were assumed to be soft faults of the lightning node site:

- The error of the soft-fault node in observing the lightning signal obeys $E\sim N(\mu ,\sigma^{2})$, where $\sigma >\sigma_{0}$ and $\sigma_{0}$ are the variances of the observation error of the normal node;
- The error of the soft-fault node in observing the lightning signal obeys $E\sim N(\lambda ,\sigma^{2})$, where λ>μ.

In the following, this paper will first define the proportion value of accuracy improvement (PI) caused by the OPE algorithm in lightning location results.

For a certain lightning event e, the observation distance from the observer node to this lightning is r. The positioning of the lightning by the lightning locator network using the Chan series algorithm is PChan, and the positioning of the lightning using the OPE algorithm is POPE. PI of the OPE algorithm for this lightning event e is defined in Equation (11), where the dis function is the distance between the two positions:

(11) $${PI}_{e}=dis(P_{OPE}-P_{Chan})/r$$

When the working states of each node in the lightning location network are normal, PIe obeys a fixed distribution. That is, in a statistical sense, the improvement amplitude of the OPE algorithm for lightning positioning accuracy should be within a certain interval. However, when there is a soft fault in one or several ordinary positioning nodes in the lightning location network, according to the definition of soft faults, the variance of the observation error dRi of each node increases, and the statistical distribution of PI deviates from the statistical distribution under the normal state.

Thus, based on the change in the PI distribution, it is possible to determine whether there are soft-fault nodes in the current lightning locator, thereby achieving the diagnosis of nodes with soft faults. This paper proposes Algorithm 2 (OPSFD) as the diagnostic algorithm, as described below.
**Algorithm 2:** OPSFD: Soft-Fault Diagnosis for Lightning Locators Based on Observer Pattern**Input:**
     **Lightning Location Network:** Composed of k-1 ordinary lightning locator nodes (N_1_, …, N_k−1_) and 1 observer lightning locator node N_0_
     **N:** lightning events detected by the lightning location network up to now    
τ: Each group has a τ lightning event
    
δ: Each segment has
δ
lightning event groups
    
**λ:** Threshold used to diagnose whether soft-fault nodes exist in a subnet
**Output**: 
    Identified ordinary lightning locator node with soft fault
**1.** **Phase 1: Statistics**
**Steps:**
    **1. Construct subnetwork:** For each ordinary node i, exclude it from lightning location network, and use the remaining nodes to form a new sub lightning location network N_i_.
    **2. Get lightning event groups:** Split historical observed lightning events into groups, each group has a predetermined number (τ) of lightning events.
    **3. Calculate PI values:** For each lightning event in the groups, the PI value of each sub lightning location network is calculated.
    **4. Calculate the variance V_i_ of PI value:** For each lightning event group, calculate the variance of PI value for sub lightning location network N_i_
**Stage 2: Diagnosis**
**Steps:**
    1**Get Segments:** Split the event groups into segments, each segment has a predetermined number (δ) of event groups.    2**Count the times of the minimum V_i_ for N_i_:** Count the times of the minimum V_i_ for each sub lightning location network N_i_ in a segment.    3**Judge:** If the number of times the minimum PI variance of subnetwork N_i_ in the segment exceeds the threshold (λ), it is judged that node i has a soft fault.


As mentioned above, the diagnostic algorithm in this study is applicable to a situation in which only one positioning node has a soft fault in the current lightning location network. The OPSFD algorithm used in this study was divided into two stages:

First stage: All ordinary lightning locator nodes are traversed. We exclude node i and use the remaining nodes to form a new lightning locator subnet Ni. Next, the historical observation data were divided into event groups according to the predetermined number of events (τ). For the lightning events within each group, the OPE algorithm is executed to calculate the PI value of each subnet for the same lightning event and the variance Vi of the PI value of each subnet Ni for each lightning event group.

Second stage: The event groups are segmented into sections according to a predetermined number of groups (δ). For each section with δ event groups, it counts the number of times the minimum Vi for each sub-lightning location network Ni is in a segment.

From the above OPSFD algorithm and the definition of a soft fault in this paper, it can be concluded that when a node has a soft fault, the distribution parameters μ and σ of the observation error of the node definitely increase. Therefore, the variance of the observation error dR = [dR0, dR1, …, dRn] of subnets, which has the soft-fault node i, will increase, whereas the observation error dR of subnets excluding the soft-fault node i remains normal.

In this paper, it is assumed that there is only one soft-fault node in the lightning location network. From the definition of the PI value, it can be obtained that Vi (the variance of the PI value) of the normal subnet Ni is the minimum.

## 4. Experiment and Analysis

This study verifies the soft-fault diagnostic algorithm for a lightning locator through simulation experiments. The diagnostic algorithm used in this study was based on the OPE algorithm. Therefore, this study first verified the improvement effect of the OPE algorithm on positioning accuracy through simulation experiments.

In the simulation experiment, the lightning location network set in this study was composed of three ordinary lightning locators (N_1_, N_2_, N_3_) and one high-precision lightning locator (N_0_), and the high-precision lightning locator was taken as the observer.

According to the existing properties of lightning locators, the positioning error of a general ordinary lightning locator is approximately 5–50 km, while that of a high-precision lightning locator is approximately 5–50 m. Thus, in the simulation experiment of this paper, the positioning error distribution of the ordinary locators is set to follow a normal distribution with μ = 10 km and *σ*^2^ = 10 km, while the positioning error distribution of the high-precision lightning locator follows a normal distribution with μ = 0.1 km and *σ*^2^ = 0.1 km.

Three types of Chan series algorithms were implemented in the simulation experiment of this study: the WLS, ML, and Chan algorithms. At the same time, based on these three types of algorithms, the OPE algorithm proposed in this paper was implemented and is named Chan*, WLS*, and ML* in the following text. In the simulation experiment, the number of candidate points for the OPE algorithm was set to 7200 points.

This study is the first to verify the effectiveness of the OPE algorithm. Through simulation experiments, the positioning results of the Chan series algorithms and the OPE algorithm implemented based on these algorithms were compared to verify the improvement effect of the OPE algorithm on lightning positioning accuracy. In the simulation of the OPE algorithm in this study, the following steps were adopted:

- The observation errors of the high-precision lightning locator, such as the mean and variance, were preset.
- The lightning occurrence positions were randomly generated within the lightning location network range. The Chan series algorithm was used to locate the observed positions of the lightning based on the observation results of the lightning location network (similar to the current coastal lightning location network using various types of lightning locator, both high-precision lightning locator nodes and ordinary lightning locator nodes were used to realize the location of lightning), and the location error was calculated.
- Defined by the OPE algorithm, lightning was first located using the Chan series algorithm based on the observation values of three ordinary lightning locators. Then, combining the location data of ordinary lightning locators with the observation values of the high-precision lightning locator, the location of the lightning was determined, and the observation error of the OPE algorithm was calculated.
- The above simulation process was repeated 100 times, and the average observation errors of different Chan series algorithms and the corresponding OPE algorithms were calculated.

The results of the OPE simulation experiments are presented in Table 1 and Table 2, where μ* is the mean of the observation error of the observer node and σ^*2^ is the variance of the observation error of the observation point node. E and E* represent the observation errors of the different algorithms (in km units). In the table, WLS/WLS*, ML/ML*, and Chan/Chan* respectively represent the estimated E and E* based on the original algorithm and the application of the OPE algorithm to the original algorithm.. 

It can be seen from the Table 1 that for different Chan algorithms, the OPE algorithm proposed in this study can significantly improve the lightning positioning accuracy of the original algorithm, with an average improvement of 25.68%, thus laying the foundation for the subsequent soft-fault node diagnostic algorithm.

The experimental results also show that for the same Chan algorithm, the improvement in the observer node positioning accuracy has little influence on the positioning accuracy of the OPE algorithm. This is because when the positioning accuracy of the ordinary lightning locator and the high-precision lightning locator differs greatly, the accuracy of the OPE algorithm still depends mainly on the accuracy of the ordinary lightning locator.

It is also worth noting that the above experimental results indicate that the OPE algorithm in this study is a low-cost and efficient method for upgrading existing lightning locator networks. By adding a high-precision locator to the existing lightning locator network and adopting the OPE algorithm, a significant improvement in the positioning accuracy of the existing lightning location network can be achieved with the condition of limited cost expenditure.

To verify the distance differences of lightning positioning between the OPE algorithm and the Chan series algorithms in the lightning positioning instrument network under the normal state (i.e., the observation errors of each node remain unchanged), this study carried out 100 simulation experiments for the three Chan series algorithms and the corresponding OPE algorithms. The experimental results are presented in Figure 3.

It can be seen from Figure 3 that the distance difference distributions of the lightning locations of the OPE and Chan series algorithms are all within a certain range, so the accuracy of the principle of the diagnostic algorithm in this study was verified.

It also can be observed from Figure 3 that the distribution range of the location distance difference of the Chan and OPE algorithms is more concentrated. Therefore, in the following, we implement the OPE algorithm based on the Chan algorithm to verify the influence of soft faults on the location difference between the original Chan and OPE algorithms.

In order to verify the diagnostic ability of the proposed OPSFD algorithm, this paper simulates the experiment based on the lightning location network of the observer mode shown in Figure 2. As shown in Figure 2, the lightning location network consists of a total of four nodes, where U is a high-precision lightning locator, and S0, S1, and S2 are ordinary lightning location.

In this paper, according to the OPSFD algorithm, the above lightning location network is divided into three subnetworks, Sub1 (S1, S2, U), Sub2 (S0, S2, U), and Sub3 (S0, S1, U), and S2 is designated as the soft-fault node. In order to simulate the influence of a soft fault on S2’s lightning location data, this paper sets a total of 14 different observation error distributions for the S2 node. For example, 8N in the second row of Table 3 shows that the observation error distribution of the S2 node caused by a soft fault is N($8\mu ,8\sigma^{2}$), where ($\mu ,\sigma^{2}$) is the observation error distribution under a normal situation.

For each soft-fault setting of the S2 node, the OPSFD algorithm is used to perform diagnosis of soft-fault nodes. In the simulation experiment, for each soft-fault setting, 500 lightning events are simulated in this paper, and 5 lightning events are regarded as an event segment. Thus, for each soft failure of the S2 node, there are 100 event fragments. For each event segment, the OPSFD algorithm is executed, and the variance of the accuracy improvement obtained by the OPE algorithm for each subnet of each event segment is counted. According to the OPSFD algorithm, the V_i_ value of each subnet in the event group is calculated in this paper, and the number of times that each subnet obtains the minimum V_i_ value in these 100 event groups is counted.

Finally, the simulation verification results of the proposed OPSFD algorithm are shown in Table 3.

As shown in Table 3, with an increase in the observation error distribution of the S2 node, which is caused by a soft fault, the number of times the smallest Vi is obtained by Sub3 (the subnet not including the faulty node S2) shows a steadily increasing trend. When the soft-fault distribution is 5N (five times the distribution parameter under normal circumstances), the number of times the smallest Vi is obtained by Sub3 exceeds half. When the soft-fault distribution is 8N, it is obvious that the anomaly in the variance of the OPE accuracy improvement is exhibited by Sub3 and the other subnets. Therefore, the above simulation experiments fully verify the effectiveness of the OPSFD algorithm in this study.

## 5. Conclusions

This paper analyzes the main factors contributing to the error of the lightning location algorithm in the application scenario of coastal lightning location, provides the error estimation of each ordinary lightning locator node in the observer pattern with the participation of observer nodes, and accordingly proposes the OPE algorithm. Because the occurrence of soft faults in ordinary locator nodes will have an impact on the improvement in the accuracy of the OPE algorithm, the node soft-fault diagnostic algorithm proposed in this paper is finally proposed.

This study verifies the improvement of the OPE algorithm in terms of accuracy through simulation experiments, thereby proving the effectiveness of the OPE algorithm. At the same time, the experiments also show the stability of the OPE algorithm in improving the positioning accuracy. Finally, the experimental results in Table 3 indicate the effectiveness of the diagnostic algorithm used in this study. In addition, it can be seen from Table 3 that the number of times Sub2 has the smallest variance in the improvement of OPE accuracy is always the least, which should be related to the spatial node positions of each ordinary node set in this simulation experiment.

In the application scenario of a lightning locator, the interference of the lightning locator may come from many aspects. In addition to Gaussian noise interference, the proposed OPE algorithm may be affected by other disturbances, which may lead to diagnostic errors.

Therefore, the proposed OPSFD algorithm first segments the lightning historical observation data according to time, and then groups them. Based on the grouped data, the OPE algorithm is executed, and the results are statistically analyzed and diagnosed. Therefore, the diagnosis results of OPSFD in this paper are based on the grouping of lightning location data to avoid misdiagnosis caused by various types of interference as much as possible.

Also, because the OPSFD algorithm is based on the statistics of a large number of lightning events, there is a lag in soft-fault diagnosis. Next, the minimum variance distribution of the accuracy improvement of each subnet in the OPSFD algorithm was studied to improve the timeliness of the diagnosis algorithm.

## Figures and Tables

**Table 1:** Comparison of lightning positioning accuracy of OPE and Chan series algorithms under different mean settings of observation error of observer nodes.

Algorithms	E/E* (μ* = μ/5)	E/E* ( μ* = μ/10)	E/E* (μ* = μ/20)	E/E* (μ* = μ/50)	E/E* (μ* = μ/100)
Chan/Chan*	17.21/13.54	16.12/13.45	17.67/12.13	18.05/13.34	19.08/14.46
WLS/WLS*	19.71/13.56	18.78/16.25	19.90/14.17	21.67/17.05	20.13/14.19
ML/ML*	15.89/11.86	17.53/14.54	17.01/14.70	17.34/14.67	18.32/15.19

**Table 2:** Comparison of lightning positioning accuracy of OPE and Chan series algorithms under different settings of observation error variances of observer nodes.

Algorithms	E/E* ($\sigma^{*2}\;=\;\sigma^{2}$/5)	E/E* ($\sigma^{*2}\;=\;\sigma^{2}$/10)	E/E* ($\sigma^{*2}\;=\;\sigma^{2}$/20)	E/E* ($\sigma^{*2}\;=\;\sigma^{2}$/50)	E/E* ($\sigma^{*2}\;=\;\sigma^{2}$/100)
Chan/Chan*	18.54/14.18	18.27/12.45	19.48/13.06	18.66/12.71	20.18/12.41
WLS/WLS*	17.17/14.34	19.10/12.28	17.39/11.05	17.13/11.24	18.60/12.25
ML/ML*	15.82/12.38	17.21/13.27	15.58/11.37	17.14/13.70	19.12/13.24

**Table 3:** Results of the simulation experiment for OPSFD diagnosis.

Sub Nets	2N	3N	4N	5N	6N	7N	8N	9N	10N	20N	30N	40N	50N
Sub1	48	44	39	37	42	35	31	26	26	30	28	16	12
Sub2	24	20	18	3	10	11	9	8	8	9	5	2	2
Sub3	28	36	43	60	48	54	60	66	66	61	67	82	86

## Data Availability

The data presented in this study are available on request from the corresponding author due to privacy concerns.
